# Medical Items, News, and Gossip

**Published:** 1871-01

**Authors:** 


### Medical Items, News, and Gossip.

Occasional bad effects of Chloral are being chronicled. A fatal case occurred recently at Guy's Hospital. Dr. Habershon commenting on it, said that it confirmed his opinion that Chloral, through its action on the pneumogastric nerve, tended to produce bronchial and pulmonary congestion. When there is any marked predisposition to paralysis, its use is apparently hazardous.——The *Med. Record* has an account of that rare form of disease, hydrocele in the female. The case was at first mistaken for inguinal hernia. The tumor gained the size of a turkey's egg. The contents of the sac were evacuated, and tincture of iodine injected, with complete cure.——It is recorded that continued external use of Carbolic Acid endangers nephritis, unless care is exercised. The urine often assumes a dark, almost tarry, appearance, with slight traces of albumen, but no blood. The dark appearance is supposed to be due to some of the oxidized products of the acid. The facts observed suggest careful observation of the urine when Carbolic Acid is used, either externally or internally.——The Boston City Hospital reports fair success in the treatment of old recurring ulcers, by transplantation of the skin.——A recent analysis of the water of the Nile shows it to be rich enough in saline matters to equal a first class magnetic spring. Incidentally from the same paper we learn that the Nile carries into the Mediterranean 55,000,000,000 tons of water each year, which certainly should make the neighborhood of that sea an excellent watering place.——At the present writing, the position of Admiral, vacated by the death of the lamented Farragut, has not been filled by the Porter who has borne so much evil and humiliation to the medical staff of the navy. Professionally we recommend our naval brethren not to advise Porter for "those who go down to the sea in ships" of war. It is singularly productive of flatulency.——Massachusetts declines hereafter to send delegates to the Am. Med. Association.——Surgeon Woodward, of the Surgeon General's staff, after carefully repeating many of Cohnheim's experiments, gives the opinion that Cohnheim was a conscientious observer, who had described as faithfully as possible the impressions made upon him.——A writer in the *British Medical Journal* is confident that fever germs are especially liable to be conveyed in *milk.* The contagion of scarlatina and the origin of enteric fever, he thinks, often bear the explanation of milk poisoning, as this fluid, from the oil particles it contains, is powerfully absorbent. It rapidly acquires odoriferous principles to which it is exposed.——Dr. Hale, of Ky., reports in the *Practitioner* a case of cure of trismus nascentium under Calomel alone. The child was six days old. Two grains of Calomel were given every two hours until the bowels were freely moved, and then repeated every eight hours. ——Warm water injections are highly recommended by Prof. Albansee, in the radical cure of hydrocele.——The best test for the purity of Hydrate of Chloral, is said (*Med. Reporter*) to be a concentrated solution of potash. The pure hydrate does not color this at all, or only a feeble yellow, and gives forth the pure smell of Chloroform. If a brown color, and the smell of Chloro-acetic Acid, or other pungent gases, be developed, the article is impure and unfit for use.——*Lusus Naturæ.* A man (?) calling himself a physician (probably the "retired," "whose sands of life had nearly run out"), is now soliciting the alms of the charitable in this city, basing his claims upon his ill health occasioned by "prolapsus uteri." Can he be the brother of the woman who sometime since was pronounced by a Chicago "physician" to have "disease of the prostate gland"?——Dr. C. B. Taylor, of New York, sues Andrew Campbell for a fee of $125 for curing his son of some difficulty not stated. The parent is recalcitrant because the Doctor brought about recovery by some other method—not employing the "Swedish Movement Cure," as defendant states he agreed to do. The *Reporter* makes no comment.——Seventy-four thousand doctors in the United States—an increase of two thousand a year since 1860. The *Reporter* thinks the American people are mulcted in $100,000,000 a year for sickness, and this without computing the quack medicines sold and swallowed—at least $25,000,000 more.——The really fair looking occupant of a chair in one of the medical colleges of this city, has been most atrociously caricatured by a lithograph in one of the advertising periodicals—called (*lucus a non lucendo*) "The Arts." It is understood that the worthy victim declines to prosecute, because in a biographical sketch, given in the same paper, he is spoken of in complimentary terms, and the writer, with admirable *naivete*, characterizes the lithograph as a good likeness, etc., etc. We tender the usual condolence.——From all accounts, the treatment of the sick and wounded in the present European war compares very unfavorably with that which was received by the same class during the recent "unpleasantness" in this country. Much complaint is made by those non-combatants willing to extend assistance, at the unfeeling, not to say brutal, manner in which their efforts are met, and the wanton waste and misapplication of supplies contributed for the relief of the unfortunate sick. Our private information coincides, fully, with what has occasionally been published on this topic by the secular press.

